# Trisulfate Disaccharide Decreases Calcium Overload and Protects Liver Injury Secondary to Liver Ischemia/Reperfusion

**DOI:** 10.1371/journal.pone.0149630

**Published:** 2016-02-22

**Authors:** Enio Rodrigues Vasques, Jose Eduardo Monteiro Cunha, Ana Maria Mendonca Coelho, Sandra N. Sampietre, Rosely Antunes Patzina, Emilio Elias Abdo, Helena B. Nader, Ivarne L. S. Tersariol, Marcelo Andrade Lima, Carlos M. G. Godoy, Tiago Rodrigues, Eleazar Chaib, Luiz A. C. D’Albuquerque

**Affiliations:** 1 Department of Gastroenterology (LIM 37), Medical School, University of Sao Paulo (USP), Sao Paulo, Brazil; 2 Department of Biochemistry, Federal University of Sao Paulo (UNIFESP), Sao Paulo, Brazil; 3 Department of Science and Technology, Federal University of Sao Paulo (UNIFESP), Sao Paulo, Brazil; 4 Center of Natural and Human Sciences, Federal University of ABC, Sao Paulo, Brazil; IDIBAPS - Hospital Clinic de Barcelona, SPAIN

## Abstract

**Background:**

Ischemia and reperfusion (I/R) causes tissue damage and intracellular calcium levels are a factor of cell death. Sodium calcium exchanger (NCX) regulates calcium extrusion and Trisulfated Disaccharide (TD) acts on NCX decreasing intracellular calcium through the inhibition of the exchange inhibitory peptide (XIP).

**Objectives:**

The aims of this research are to evaluate TD effects in liver injury secondary to I/R in animals and *in vitro* action on cytosolic calcium of hepatocytes cultures under calcium overload.

**Methods:**

Wistar rats submitted to partial liver ischemia were divided in groups: Control: (n = 10): surgical manipulation with no liver ischemia; Saline: (n = 15): rats receiving IV saline before reperfusion; and TD: (n = 15): rats receiving IV TD before reperfusion. Four hours after reperfusion, serum levels of AST, ALT, TNF-α, IL-6, and IL-10 were measured. Liver tissue samples were collected for mitochondrial function and malondialdehyde (MDA) content. Pulmonary vascular permeability and histologic parameters of liver were determined. TD effect on cytosolic calcium was evaluated in BRL3A hepatic rat cell cultures stimulated by thapsigargin pre and after treatment with TD.

**Results:**

AST, ALT, cytokines, liver MDA, mitochondrial dysfunction and hepatic histologic injury scores were less in TD group when compared to Saline Group (p<0.05) with no differences in pulmonary vascular permeability. In culture cells, TD diminished the intracellular calcium raise and prevented the calcium increase pre and after treatment with thapsigargin, respectively.

**Conclusion:**

TD decreases liver cell damage, preserves mitochondrial function and increases hepatic tolerance to I/R injury by calcium extrusion in Ca2+ overload situations.

## Introduction

Ischemia-reperfusion (I/R) injury remains a major problem in various clinical situations, including trauma, shock, hemorrhage, liver resection, and transplantation [1, 2]. Current therapeutic procedures, such as ischemic pre or post conditioning and intermittent vascular occlusion, can minimize but not prevent hepatic I/R injury [3–6]. Up to the present time, although many compounds have been tested, only a few drugs have been shown to ameliorate or diminish liver I/R injury [7–8].

The pathophysiology of hepatic damage after I/R is not entirely understood, but the changes in intracellular calcium behavior has emerged as an important mechanism in the development of cell damage [9, 10]. It has been shown that inhibition of cellular calcium accumulation prevents hepatic I/R damage [11]. Furthermore, expression of calcium-sensing receptors plays a vital role in apoptosis induced by I/R injury [12] and a decrease in mitochondrial Ca^2+^ levels are associated with decreased apoptosis and cellular changes [13]. In addition, inhibition of intracellular calcium overloading effectively minimizes organ damage in models of liver I/R [14]. The intracellular calcium concentration is controlled by the membrane sodium-calcium exchanger (NCX), which decreases calcium by exchanging calcium for extracellular sodium. An endogenous regulatory peptide named Exchange Inhibitor Peptide (XIP) modulates the activity of NCX [15]. In contrast, the *in vitro* stimulation of lipid peroxidation in isolated rat hepatocytes increases cytosolic calcium levels by the influx of extracellular calcium especially in the absence of sodium. When sodium is added to the incubation medium, the influx of calcium induced by lipid peroxidation diminishes, preventing cell death, this process being mediated by NCX [16].

Trisulfated disaccharide (TD) affects NCX action in rabbit endothelial aortic cells by decreasing intracellular calcium through the inhibition of XIP [17]. However, the TD effect in the liver has never been previously investigated. Thus, the purposes of this study were to investigate the effects of the reduction of intracellular calcium concentration, determined by TD administration, on the hepatic injury of rats submitted to liver I/R and to demonstrate TD effect *in vitro* on hepatocyte cultures under calcium overload.

## Materials and Methods

### *In vivo* experiments

#### Animals

Forty *adult* male Wistar rats weighing 250-300g housed in individual cages in a 12h dark-light controlled environment were used for the experimental protocol. Rats had free access to standard rat chow and water. The experimental protocol was approved by the Ethics Committee for Animal Research of the Medical School of São Paulo University (research protocol n.138/13 in 04/24/2013) and received humanized care according to the criteria outlined in the Guide for the Care and Use of Laboratory Animals (Institute of Laboratory Animal Resources, Commission on Life Sciences and National Research Council. National Academic Press, Washington, D.C., 1996).

#### Experimental design

The animals were randomly submitted to the following experimental protocols:

*Control Group* (CONTR): 10 animals submitted to surgical liver manipulation with no hepatic ischemia induction.
*Saline Group* (SAL): 15 animals submitted to hepatic ischemia for one hour that received 0.4 ml of saline solution (0.9% NaCl) injected via the dorsal penile vein 10 minutes before liver reperfusion.
*Trisulfated disaccharide Group* (TD): 15 animals submitted to hepatic ischemia for one hour that received 0.2 mg/kg of trisulfated disaccharide (UNIFESP, Molecular Biology Laboratory, São Paulo, Brazil) diluted in 0.4 ml of 0.9% NaCl, injected via the dorsal penile vein 10 minutes before liver reperfusion.

#### Surgical procedure

Animals were anesthetized intraperitoneally with 30mg/kg of ketamine hydrochloride (Cristália, São Paulo, Brazil), and 30 mg/kg of xylazine (Bayer, São Paulo, Brazil), and kept under mechanical ventilation (Small Animal Ventilator model 683, Harvard Apparatus, Holliston, MA, USA) at a frequency of 60 cycles per minute and a tidal volume of 0.08 ml/kg. Animal’s body temperature was monitored throughout the whole procedure by a digital rectal thermometer (YSI Precision 4000A Thermometer, USA) and kept between 35 and 37°C through external heating by a halogen lamp (45 W and 127 V). The surgical procedure was performed through a 4 cm laparotomy extending caudally from the xiphoid process with dissection of the medium and lateral common hepatic pedicle in all the three experimental groups. Partial liver ischemia (70% of liver parenchyma) was produced in the SAL and TD groups for one hour by clamping the pedicles of the median and left lateral hepatic lobes. The abdominal incision was closed and reopened after 60 min for clamp removal and liver reperfusion [18].

#### Sample collection

At 4 h after reperfusion, the animals were re-anesthetized as described previously and submitted to laparotomy for collection of blood and tissues according to the study design. The 4-hour period after the reperfusion has been chosen based on the results of several previous studies from our laboratory that could demonstrate a significant major hepatic damage at this time period compared to more prolonged time observation periods [19, 20]. Blood was collected by transdiaphragmatic cardiac puncture for determinations of AST, ALT, TNF-α, IL-6 and IL-10. Euthanasia was performed by transecting the abdominal aorta and inferior vena cava. Liver tissue was removed and samples of the ischemic lobes were obtained for assessments of mitochondrial oxidation and phosphorylation, malondialdehyde (MDA) contents, and histologic analysis. Pulmonary vascular permeability was determined in lung tissue by Evans blue (EB) dye extravasation.

#### Serum aspartate aminotransferase (AST) and alanine aminotransferase (ALT) levels

The extent of hepatocellular injury was evaluated by determination of serum AST and ALT levels. Quantification of enzyme activities was performed by optimized ultraviolet method (COBAS MIRA, Roche) and results expressed in U/L.

#### Liver mitochondrial oxidation and phosphorylation activities

Liver mitochondrial function was studied in ischemic liver lobes separated after liver removal and immersed in homogenization solution for mitochondrial extraction as described previously [21].

Mitochondrial oxygen consumption (activated state, S3 and basal state, S4) was measured polarographically [22] using a Gilson 5/6H Oxygraph (Gilson Medical Electronics, Inc., Middleton, WI) in a closed reaction vessel fitted with a Clark oxygen electrode (Yellow Springs Instruments Co., Yellow Springs, OH) at 28°C, using succinate as respiratory substrate. The ratio of oxygen consumption in the presence of ADP to that in the absence of ADP (respiratory control rate, RCR) and the ADP/O ratio were calculated as indices of oxidative and phosphorylative mitochondrial functions. [23]

RCR = oxygen consumption in state 3 / oxygen consumption in state 4.

ADP/O = moles of ATP formed from ADP per atom of oxygen consumed.

Respiratory states 3 and 4 were measured and reported as nanogram atoms of oxygen per milligram mitochondrial protein per minute. Mitochondria protein content was determined by the method of Lowry et al [24].

#### Lipid peroxidation analysis

Malondialdehyde (MDA) formation was used as indicative of the occurrence of lipid peroxidation in the tissues and was estimated as thiobarbituric acid-reactive substances (TBARS). Liver tissues (100 mg/ml) were homogenized in 1.15% KCl buffer and centrifuged at 14,000×g for 20 min. An aliquot of the supernatant was then added to a reaction mixture consisting of 1.5 ml 0.8% thiobarbituric acid, 200 μl 8.1% (v/v) sodium dodecyl sulfate, 1.5 ml 20% acetic acid (pH 3.5), and 600 μl distilled water. The mixture was then heated at 90°C for 45 min. After cooling to room temperature, samples were cleared by centrifugation (10,000×g for 10 min), and the absorbance was measured at 532 nm using malondialdehyde bis (dimethyl acetal) as external standard. The content of lipid peroxides was expressed as nmol MDA per mg of protein [25].

#### Determination of inflammatory mediators

Serum levels of TNF-α, IL-6, and IL-10 were determined by ELISA using commercial kits (Invitrogen, CA, USA).

#### Lung tissue microvascular permeability analysis

Lung microvascular permeability was quantified by the Evans blue dye (EBD) extravasation technique as described previously [26]. The results are expressed as microgram of EBD per gram of dry weight tissue. Expression of results as a function of dry weight avoids under evaluation due to edema formation.

#### Histologic analysis of the liver

Liver samples were fixed in 10% buffered formalin for standard hematoxylin and eosin staining. Histologic evaluation of the liver sections was performed by the same blinded pathologist. The severity of histologic injury was analyzed according to the scoring system proposed by Quireze et al [27].

#### Trisulfated Dissacharide

Enzymes of adapted *Flavobacterium heparinun* degraded heparin and its degradation products were separated by combined Sephadex-gel filtration, paper chromatography and chemically analysed. These products were identified as saturated disaccharides constituted of uronic acid and glucosamine and containing three sulphate residues with the same basic disaccharide units [28].

### *In vitro* studies

#### Culture cells and TD

BRL3A culture cells from *Rattusnorvegicus* liver (cell line that express NCX protein, the suggested target of TD) were grown in supplemented Dulbecco’s modified Eagle’s medium for 24h [29]. Then, were loaded with the fluorescent Ca^2+^ indicator fura-2/AM (4 μM) for 30 min at 25°C in a 5% CO_2_ atmosphere. Changes in cytosolic calcium levels were measured kinetically at 37°C for 15 min. The ratio images were obtained using Calcium Imaging mode in Leica Application Suite 3D Deconvolution & 3D Visualization software. Thapsigargin (4 μM) was added to the culture medium to promote a sustained raise in the cytosolic calcium level from endoplasmic reticulum. Calcium ionophore ionomycin (0.25 μM) was used at the end of each experiment to reach the maximal increase in cytosolic calcium. To evaluate the TD effect on cytosolic calcium level, 30 μM of TD was added to the medium 120 seconds before and after the thapsigargin addition. The experiment was repeated for three times under the same experimental conditions.

#### Statistical analysis

Results are presented as mean values ± SEM. Continuous variables were compared using analysis of variance and post hoc testing with Tukey-Kramer multiple comparison test. Results from the histologic analysis were compared using the Kruskal-Wallis test and Mann-Whitney’s test. The GraphPad 6 Prism Software (GraphPad Software, San Diego, CA) was used for statistical analysis. The level of p<0.05 was considered as statistically significant.

## Results

### Animal experiments

There were no experimental animal deaths in the present study and all animals finished the experiments successfully.

#### Hepatic enzymes (Serum AST and ALT)

**Fig 1:**
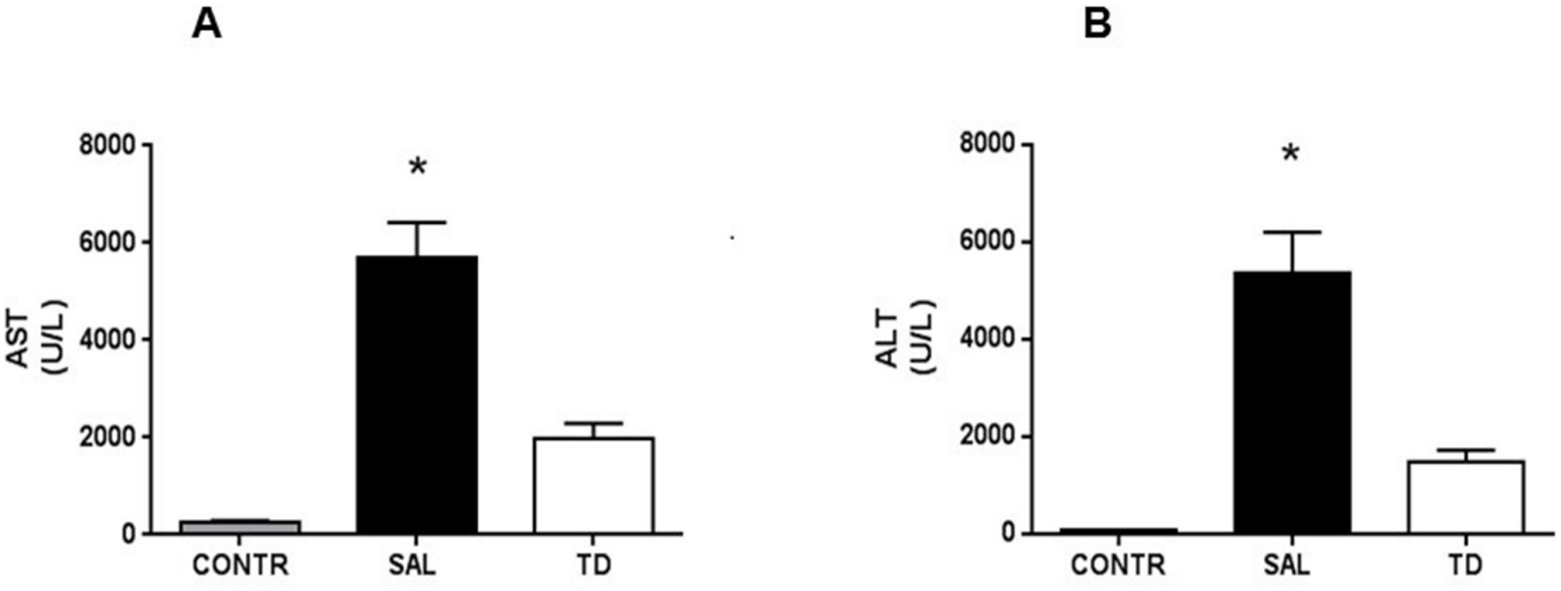
Aminotransferases four hours after liver reperfusion. 1A –AST—aspartate aminotransferase; 1B –ALT—alanine aminotransferase CONTR—Control Group (n = 10); SAL—Saline Group (n = 15); TD—Trisulfated Disaccharide Group (n = 15). Data are expressed as mean values * (SAL vs TD and SAL vs CONTR) p<0.05.

Four hours after reperfusion, serum levels of both AST and ALT were significantly increased in the SAL and TD groups when compared to the CONTR group. However, in the TD group AST and ALT serum levels were significantly lower than in the SAL group (Fig 1A and 1B).

#### Liver mitochondrial oxidation and phosphorylation activities

**Fig 2:**
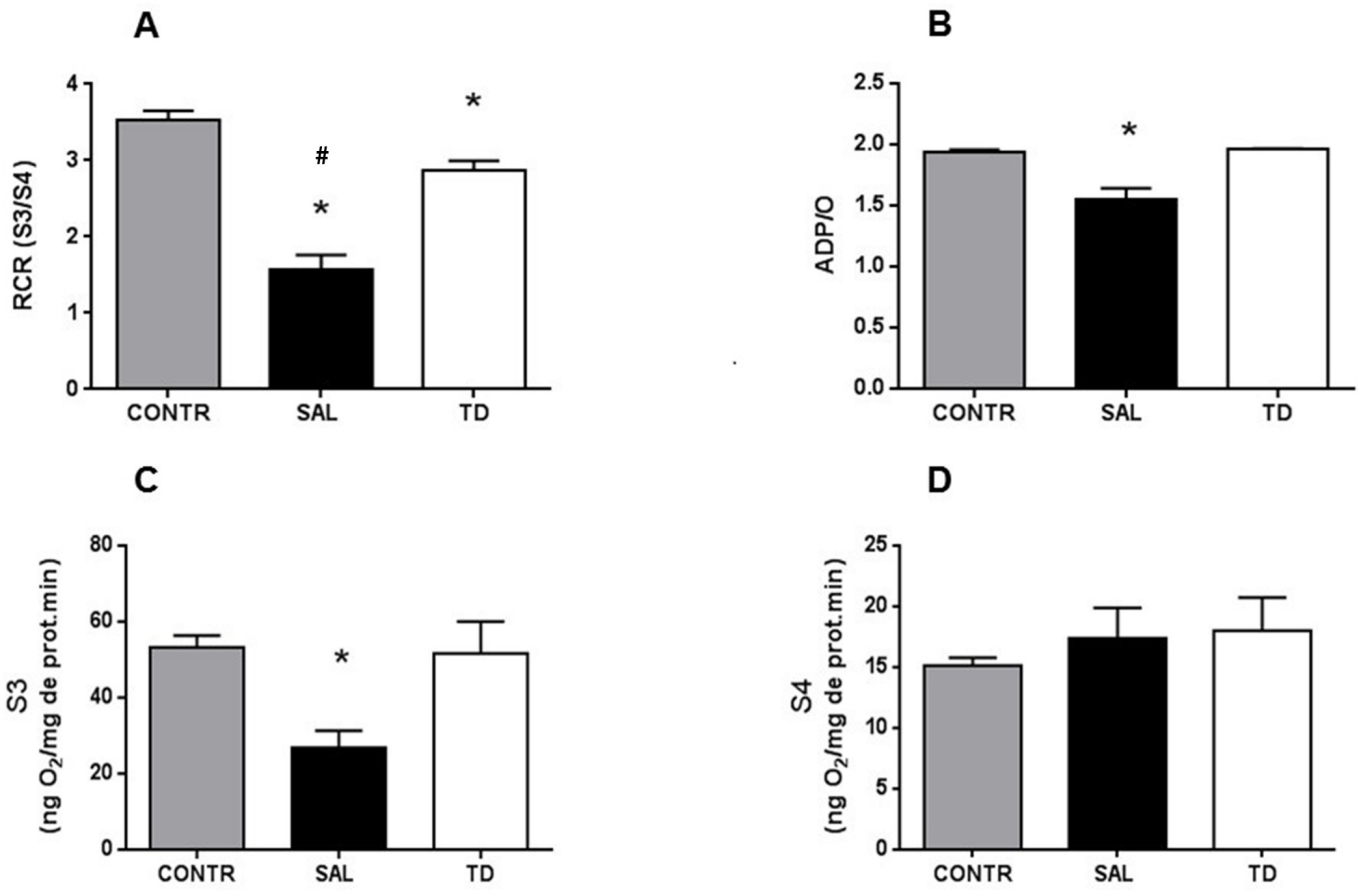
Liver mitochondrial function. 2A –RCR—Respiratory control rate * (TD vs SAL) p<0.05; # (CONTR vs SAL) p<0,05; 2B—ADP/O—ADP/Oxygen ratio * (SAL vs TD and SAL vs CONTR) p<0.05; 2C –S3—State 3 respiration * (SAL vs TD and SAL vs CONTR) p<0.05; 2D –S4—State 4 respiration NS: No Significance. CONTR—Control Group (n = 10); SAL—Saline Group; TD (n = 15)—Trisulfated Disaccharide Group (n = 15). Data are expressed as mean values.

Four hours after reperfusion, there was a decrease in the oxygen consumption rate by liver mitochondria in state 3 (S3), in respiratory control ratio (RCR), and in ADP/O ratio in the SAL group. This decrease was not observed in TD and CONTR groups. No differences were found in state 4 (S4) between SAL and TD groups (Fig 2A, 2B, 2C and 2D).

#### Liver MDA and lung permeability

**Fig 3:**
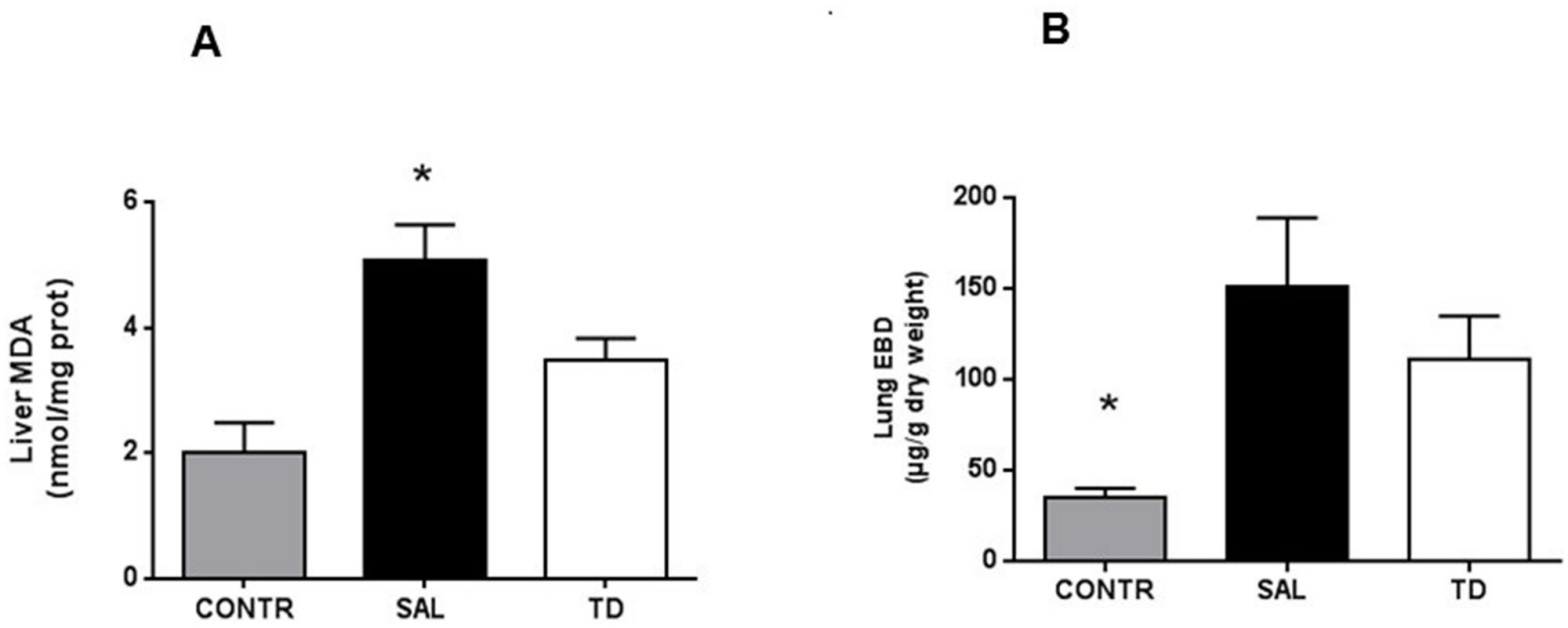
Liver MDA and Lung microvascular permeability (EBD). 3A –Liver MDA. Data are expressed as mean values * (SAL vs TD and SAL vs CONTR) p<0.05; 3B—Lung microvascular permeability (EBD) *(CONT vs SAL and CONTR vs TD) p<0.05. (TD vs SAL) NS. CONTR—Control Group; SAL (n = 10)–Saline Group (n = 15); TD—Trisulfated Disaccharide Group (n = 15). NS: No Significance.

At four hours after reperfusion, MDA content was significantly increased in the ischemic liver in the SAL group. MDA content was also increased in TD group but in a much lower extent, showing that TD significantly decreased the liver peroxidation induced by the I/R injury (Fig 3A). Lung microvascular permeability, as evaluated by EBD extravasation, was increased 4 h after reperfusion in the SAL and TD groups compared to the CONTR group, without significant difference between TD and SAL groups. (Fig 3B).

#### Inflammatory mediators

**Fig 4:**
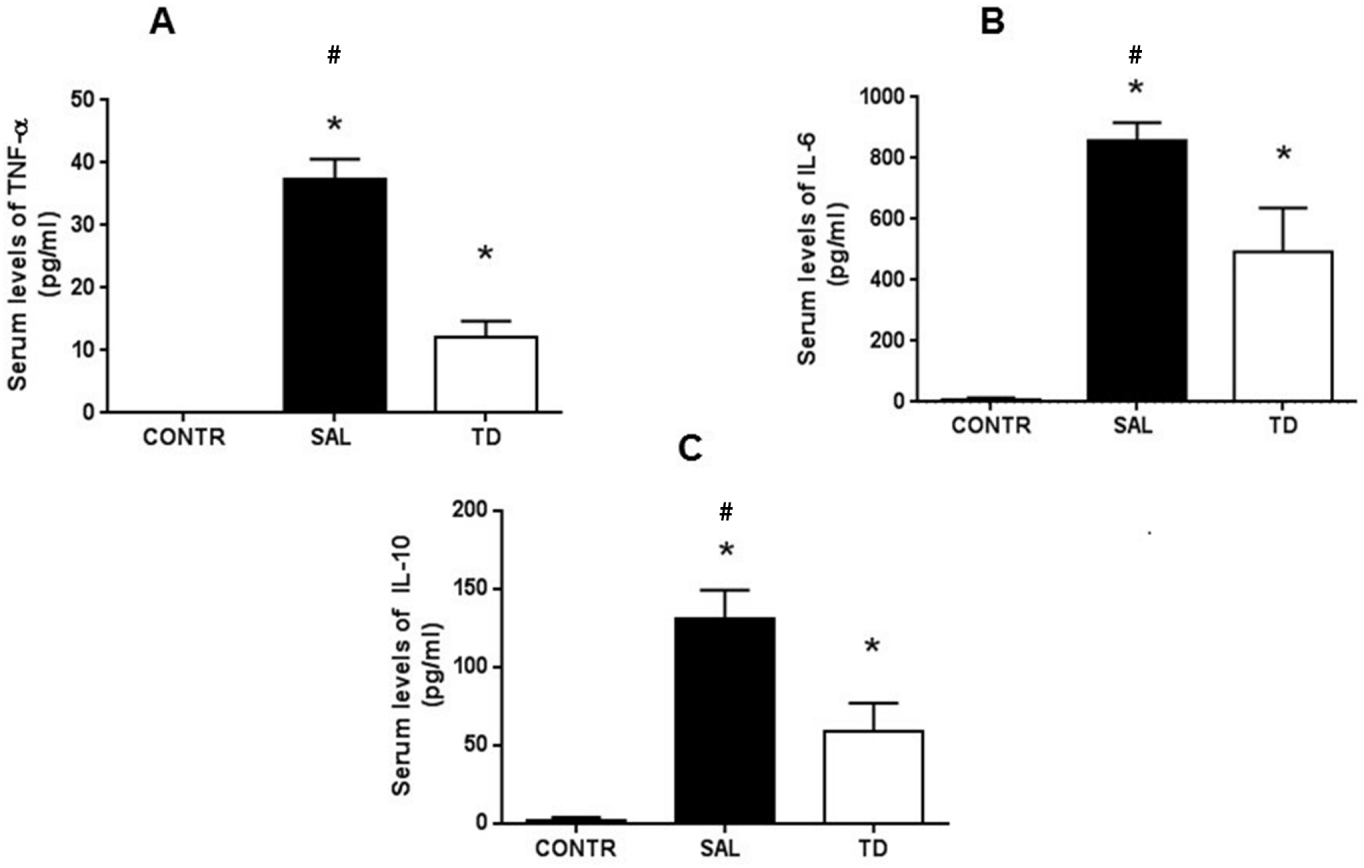
Serum levels of inflammatory mediators. 4A—TNF-α; 4B—IL-6; 4C—IL-10 * (TD vs SAL) p<0.05; # (CONTR vs SAL) p<0.05. CONTR—Control Group (n = 10); SAL—Saline Group (n = 15); TD—Trisulfated Disaccharide Group (n = 15). Data are expressed as mean values.

A significant increase in serum levels of TNF-α, IL-6, and IL-10 was observed in the SAL and TD group compared to the CONTR group. However, serum levels of all three mediators were significantly lower in TD group when compared to the SAL group (Fig 4A, 4B and 4C). As expected, TNF-αcould not be detected in the serum of animals from CONTR group.

#### Liver histologic analysis

**Fig 5:**
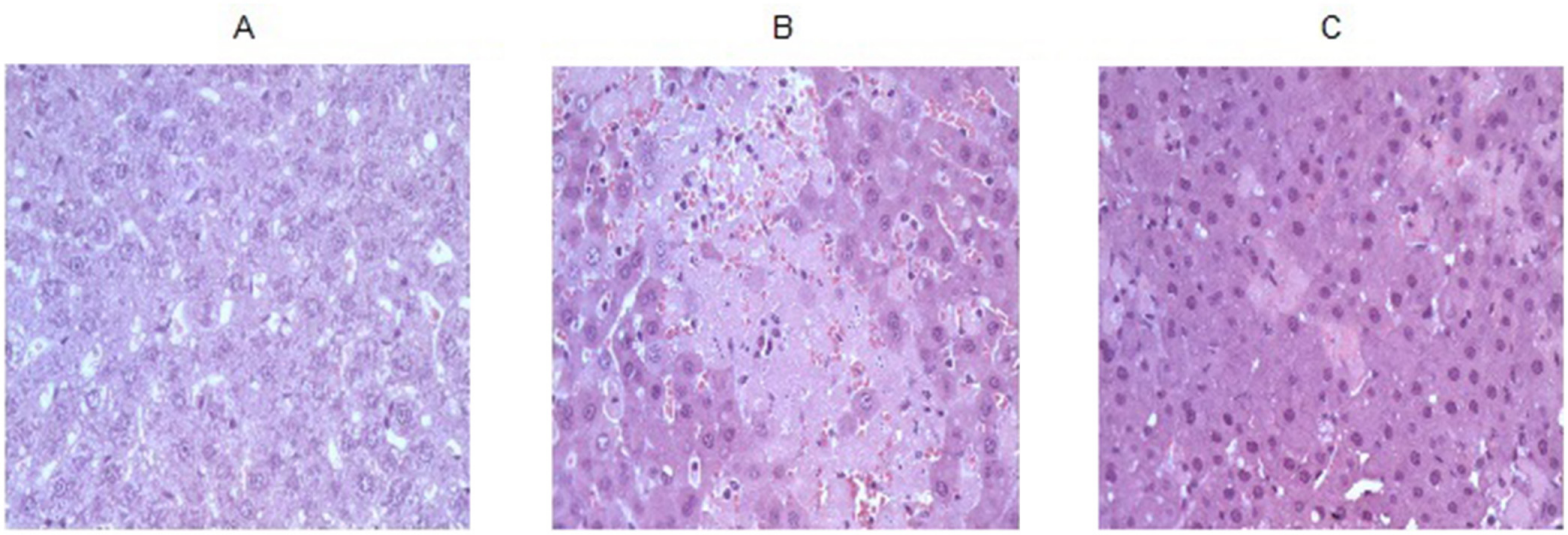
Liver histology. Liver cell necrosis was significantly lower in the TD group when compared to the SAL group. 5A– Control Group (n = 10); 5B –Saline Group (n = 15); 5C –Trisulfated Disaccharide Group (n = 15).

The histologic injury scores at 4 hours after liver reperfusion was decreased in TD group compared to the SAL group. Additionally, liver cell necrosis was significantly lower in the TD group when compared to the SAL group (Table 1 and Fig 5A, 5B and 5C).

**Table 1:** Liver histologic analysis.

Groups	Liver Histologic Score	Coagulation Necrosis Score
**Control** (n = 10)	0 (0–3)	0 (0–3)
**Saline** (n = 15)	9 (2–11) *	7.5 (0–9) *
**TD** (n = 15)	0 (0–5)	0 (0–3)

Data are presented as median and range TD—Trisulfated. Disaccharide * *p<0*.*01 vs* Control and TD groups.

### *In vitro* studies

#### Cytosolic calcium levels under TD action in liver cell cultures

**Fig 6:**
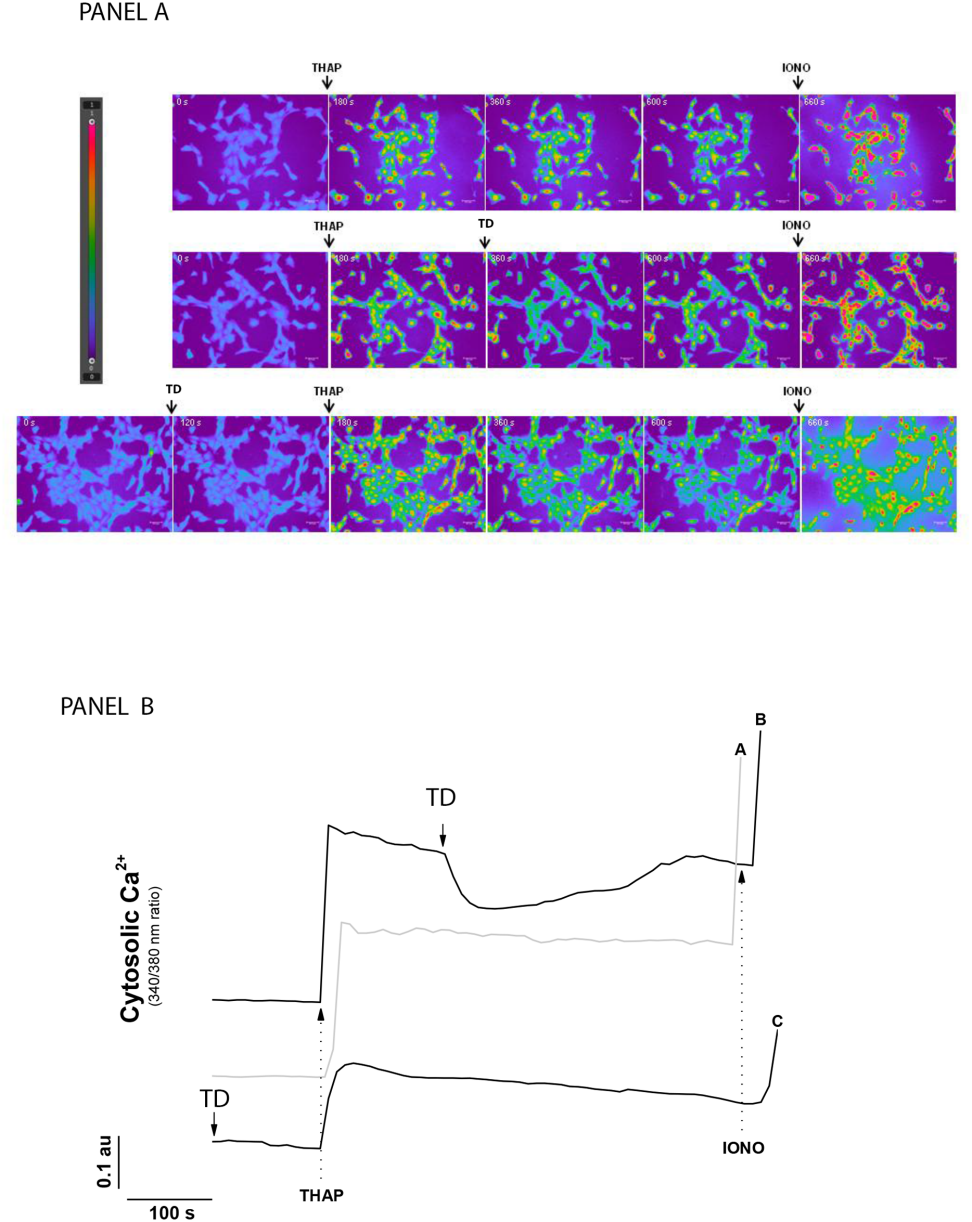
TD effect on thapsigargin-induced cytosolic calcium increase in liver cells (n = 3). Panel A—Images of 340/380 nm fluorescence ratio using a non-true color scale. Scale bars—50 μm; Time shown in white inside each frame; Panel B—Relative quantification of changes in calcium levels. Arrows indicate additions. THAP—4 μM thapsigargin; IONO—0.25 μM ionomycin; TD—30 μM trisulfated disaccharide.

Representative ratio images of BRL3A cells loaded with fura-2 are presented in Fig 6, panel A, with a scale color varying from blue (minimum) to pink (maximum). In panel B, the kinetics of the cytosolic calcium variation of each sample is shown. As expected, the addition of 4 μM thapsigargin promoted a sustained raise in the cytosolic calcium level in BRL3A liver cells (Fig 6, panel A). The addition of TD to the culture medium after the thapsigargin-induced cytosolic calcium increase transiently decreased intracellular calcium levels for about 250 seconds (Fig 6, panel B). In contrast, when TD was added prior to thapsigargin, just a slight decrease of intracellular calcium basal level was observed. However, TD addition prior thapsigargin resulted in a lesser extend of cytosolic calcium increase determined by thapsigargin. These results show that TD was able to decrease the intracellular calcium raise promoted by thapsigargin and to prevent it when added prior to the inducer.

## Discussion

Multiple clinical situations expose the liver to I/R injury. The pathogenesis of I/R damage is multifactorial [30–32]. Altered cellular calcium handling is known to be of vital importance in the I/R injury. Many investigators agree that modulation of cellular calcium homeostasis represents a key strategy for preventing I/R injury [33].

Heparin compounds have been studied in I/R injury. Heparin administration in high doses has no beneficial effects and is associated with bleeding complications in liver and kidney injury on the rat experimental model of I/R [34]. In contrast, protective effects of heparin were demonstrated on hepatic I/R lesions in rabbits [35]. The heparin fragment Dalteparin reduces I/R-induced liver injury in rats by attenuating the inflammatory response due to its capacity to enhance endothelial production of prostacyclin via cyclooxygenase-1 activation, independently of its anticoagulant activity [36]. N-desulfated heparin prevents hepatic/renal damage induced by I/R injury without significant anticoagulant activity [37]. Enoxaparin has been shown to decrease hepatic necrosis in a rat model of cholestatic liver injury, but this same effect was not observed with heparin or tinzaparin [38]. The effect of different heparins on calcium extrusion depends on the correlation between the fragments molecular weight and calcium modulation [17]. Therefore, heparin compounds might be useful in liver protection but bleeding represents a limiting factor in the eventual clinical use.

TD is the smallest heparin fragment, 585 Da weight, with no anticoagulant effect and higher affinity to XIP than other heparin fragments [17]. It probably increases the activity of NCX in the liver tissue by inhibition of XIP, thus accelerating calcium extrusion in situations of calcium overload in a similar way as observed in muscle cell lines [17]. Therefore, it appears that XIP, a part of NCX structure, is the target for the effect of TD in the toxic accumulation of calcium that occurs during ischemia. Thus, it may have an eventual application in the clinical setting to protect cells in calcium overload situations.

The present study, based on a model of partial warm hepatic I/R injury, showed that TD pretreatment decreases hepatic I/R injury as evidenced by diminished AST and ALT levels as well as by attenuated histopathological alterations. Furthermore, TD pre-treatment suppressed the increases of inflammation serum markers (TNF-α, IL-6, IL-10) and hepatic lipid peroxidation associated with hepatic lesion after I/R. In addition, mitochondrial function was preserved under this situation. The protective mechanism involves the action of TD on intracellular calcium extrusion, as demonstrated in the Fura-2 *in vitro* experiments, since TD was able to decrease the intracellular calcium raise promoted by thapsigargin and to prevent it when added prior to the inducer, suggesting a better protection in the pretreatment setting. These findings are in agreement with the results observed *in vivo*, when liver protection was observed when TD infusion was performed 10 minutes before reperfusion.

In smooth muscle cell and, in other cell lines with NCX expression, TD decreases intracellular calcium in a dose-dependent manner via its binding to NCX [17]. In a human hepatoma cell line, it was proposed that *tert*-butyl hydroperoxide induced apoptosis and increased intracellular calcium concentration through the activation of an inhibitory mechanism on NCX [39].

The Inflammatory cytokines TNF-α and IL-6 and, the anti-inflammatory IL-10 are released after I/R injury [11]. Several reports indicate that Kupffer cells are activated during ischemia/reperfusion injury increasing the production of cytokines and ROS [40, 41]. In the present study, a significant decrease of serum levels of cytokines in the TD treated animals was observed (Fig 4A, 4B and 4C). This may reflect a reduction of hepatic lesion after the ischemia/ reperfusion injury. However, other inflammatory triggers, such as damage-associated molecular patterns (DAMPs), that are released from necrotic cells, may also contribute to inflammation associated with hepatic ischemia-reperfusion injury [42,43]. Excessive ROS signaling leads to cell death after I/R and, similarly to the ischemic accumulation of succinate, calcium also participates in the reactions responsible for an extensive ROS generation [44, 45].

An anti-inflammatory effect of the low molecular weight heparin enoxaparin was described on carbon tetrachloride induced hepatic necrosis and apoptosis in the liver of rats [46]. In the present study, TD pretreatment also suppressed the increases of TNF-α, IL-6, and IL-10 serum levels that occur after hepatic I/R, suggesting that modulation of the inflammatory response could be another mechanism of TD liver protection. This anti-inflammatory effect appears to be a local and immediate effect of TD since no distant benefit, as measured by pulmonary vascular permeability, was obtained after the four-hour observation period in this study. The decrease of IL-10 levels might be explained by the diminished cell damage determined by the TD pretreatment.

Lipid peroxidation, as evaluated by MDA, is a mechanism of cell injury mediated by reactive oxygen species (ROS). Both excessive ROS signaling and calcium overload lead to cell death after I/R as calcium participates in the reactions that generate ROS [44, 45]. Our findings showed that MDA levels in the ischemic liver lobe of TD pretreated animals were lower than saline treated rats also suggesting a liver protective effect of TD. This observation is consistent with the histologic observations that showed much less necrosis after I/R in the TD group. The sustained increase in cytosolic calcium is commonly accompanied by mitochondrial calcium uptake. Such increased mitochondrial calcium uptake results mitochondrial membrane permeabilization leading to caspase activation and disruption of multiple cellular functions [47]. Our assessment of mitochondrial function revealed the maintenance of oxygen consumption rates in TD animal group comparable to the control group and higher than the saline group. This mitochondrial protective effect of TD, also suggested by the histological findings, is possibly related to calcium extrusion via NCX.

Animal models are frequently used to study possible pharmacological agents to attenuate I/R in liver transplantation. A recent systematic search of the PubMed database for literature in which pharmacological agents against I/R injury have been studied using rat liver transplant models showed, by the results of a meta-analysis, that pharmacological treatment is effective to reduce I/R liver damage in hepatic transplantation [48]. Based on the experimental data that are available today, the authors propose that identified agents should be further evaluated in human liver transplantation. Considering the findings of the present study TD represents a promising agent that might be considered for evaluation in human liver protection. Although this rat I/R model may not be completely translatable to human clinical characteristics of hepatic I/R [49], it was purposely used in the present study with the intend of testing the eventual protective effect of TD under extreme liver damage conditions. In conclusion, the results of this study demonstrate that trisulfated disaccharide pretreatment in a rat model of liver I/R injury achieves hepatic protection, probably via modulation of intracellular calcium concentrations mediated by Na-Ca exchanger. This hypothesis was demonstrated by the acceleration of calcium extrusion and decrease of calcium levels in liver cell cultures pretreated with DT. More data are necessary to further elucidate the mechanisms involved before its application in the clinical setting is justified.
